# Pixelated Microfluidics for Drug Screening on Tumour Spheroids and Ex Vivo Microdissected Tumour Explants

**DOI:** 10.3390/cancers15041060

**Published:** 2023-02-07

**Authors:** Dina Dorrigiv, Pierre-Alexandre Goyette, Amélie St-Georges-Robillard, Anne-Marie Mes-Masson, Thomas Gervais

**Affiliations:** 1 Centre de Recherche du Centre Hospitalier de l’Université de Montréal (CRCHUM), Institut du Cancer de Montréal, Montreal, QC H2X 0A9, Canada; 2 Institute of Biomedical Engineering, Polytechnique Montréal, Montreal, QC H3T 1J4, Canada; 3 Department of Engineering Physics, Polytechnique Montréal, Montreal, QC H3T 1J4, Canada; 4 Department of Medicine, Université de Montréal, Montreal, QC H3T 1J4, Canada

**Keywords:** drug development, personalized medicine, preclinical assay, 3D tumour models, spheroids, ex vivo tumour explants, open-space microfluidics

## Abstract

**Simple Summary:**

A major challenge in the treatment of cancer is predicting patients’ responses to anticancer drugs. Thus, preclinical assays that reflect patients’ responses to treatments are of utmost importance in clinical oncology and in developing new drugs. 3D tumour models such as spheroids and ex vivo tumour explants are appropriate preclinical models. However, the short-term longevity and low throughput of these models limit their application. To address this, we present a computer-controlled drug screening platform that enables multiplexed delivery of several biochemical reagents such as cellular dyes to 3D tumour models. The platform enables testing up to nine distinct treatment conditions (i.e., nine different biochemical reagents) on more than a hundred 3D tumour models. Moreover, it is compatible with clinical histopathology practice for further manipulation and treatment response analyses of tumour models.

**Abstract:**

Anticancer drugs have the lowest success rate of approval in drug development programs. Thus, preclinical assays that closely predict the clinical responses to drugs are of utmost importance in both clinical oncology and pharmaceutical research. 3D tumour models preserve the tumoral architecture and are cost- and time-efficient. However, the short-term longevity, limited throughput, and limitations of live imaging of these models have so far driven researchers towards less realistic tumour models such as monolayer cell cultures. Here, we present an open-space microfluidic drug screening platform that enables the formation, culture, and multiplexed delivery of several reagents to various 3D tumour models, namely cancer cell line spheroids and ex vivo primary tumour fragments. Our platform utilizes a microfluidic pixelated chemical display that creates isolated adjacent flow sub-units of reagents, which we refer to as fluidic ‘pixels’, over tumour models in a contact-free fashion. Up to nine different treatment conditions can be tested over 144 samples in a single experiment. We provide a proof-of-concept application by staining fixed and live tumour models with multiple cellular dyes. Furthermore, we demonstrate that the response of the tumour models to biological stimuli can be assessed using the platform. Upscaling the microfluidic platform to larger areas can lead to higher throughputs, and thus will have a significant impact on developing treatments for cancer.

## 1. Introduction

A major impediment to cancer treatment is predicting the response of patients to anti-cancer drugs as they have an extremely low clinical approval rate in drug development programs [1,2,3]. Improving preclinical models to predict the response of patients to treatments can improve drug precision and effectiveness, spare patients from exposure to unnecessary toxicities, accelerate the drug development process, and ultimately reduce healthcare costs [4,5]. Various predictive preclinical tumour models are available to researchers. Preclinical tumour models include 2D monolayer cultures of cancer cells; 3D tumour models such as cancer cell line spheroids, tumour organoids, ex vivo cultured tumour fragments; and in vivo models, from the simplest to the most complex, respectively. Monolayer cell cultures are easy to replicate but lack the 3D tumour structure and the interactions between the cancer cells and the tumour microenvironment [6]. In vivo models are the gold standard of preclinical models, but their production is time-consuming and labour-intensive. They may also fail to predict the clinical efficacy of drugs due to species differences [7]. 3D tumour models can bridge the gap between 2D and in vivo models: unlike 2D monolayers, 3D tumour models mimic the tumoral architecture and are human-derived and easier to work with than in vivo models [8,9,10,11,12,13]. Three main groups of 3D tumour models exist. In order of increasing complexity and in vivo relevance, they are cell line spheroids, tumour organoids, and ex vivo cultured tumour explants. They can be selected according to the purpose and requirements of a given study [12,14]. Drawbacks of the various 3D tumour models include limitations of live imaging and interfacing with histopathology, and the generally low throughput and low viability of tissue, especially for ex vivo tumour explants [15]. The most advanced live imaging methods, such as confocal and multiphoton microscopy, are well known to have severe limitations in 3D biology, notably their limited light penetration depth in live tissue and cost [16]. In addition, the universally recognized standard for primary tissue-based clinical decision-making is histopathology (i.e., the practice of preserving tumour tissues in paraffin or a freezing medium and dissecting them into thin (5–10 µm) slices) [17]. To overcome the limitation of live imaging and increase clinical relevance, in particular taking into account routine clinical pathology, it would be advantageous for 3D tumour models to be compatible with standard histopathology practice. Various techniques have been developed for culture and drug screening on 3D tumour models and preparing them for histopathological analyses. The most conventional technique is culturing tumour models in plastic well plates. Samples are subjected to reagents manually or using robotic liquid handlers in wells. Sample manipulation using pipettes, whether manually or using pipetting robots, imposes risks such as aspirating or shearing the sample while changing the medium. In addition, removing the samples out of the wells for further histopathology processing is tedious. Moreover, plastic well plates are not optimal for preserving the viability and metabolic activity of fragile 3D tumour models, such as ex vivo tumour explants. Our group has previously studied the ex vivo survival of tumour tissue explants and has shown that tumour tissue slices cultured in non-perfused well plates start to die in two days due to insufficient oxygen supply [18]. Microfluidics can palliate this problem by introducing chips for high-throughput processing of sub-millimetre-sized ex vivo tumour explants [6,19]. The drawback of most microfluidic chips is that they require sample entrapment in closed microchannels, and are not amenable to surface-based work environments such as Petri dishes [20].

New culture platforms that can improve survival and high-throughput drug screening on 3D tumour models, while remaining fully compatible with gold-standard tissue analysis, are promising avenues to improve pre-clinical drug testing. In this article, we present a platform that bridges the concepts behind well plates and perifusion-based microfluidics. The platform uses open-space microfluidic laminar flow confinement to stream reagents within isolated flow sub-units over a large number of various 3D tumour models. Open-space microfluidic systems are channel-free and contact-free fluidic processors that deliver reagents directly over the sample [21]. Pioneering open-space microfluidic systems have been used for various purposes including single cell analysis [22,23,24], perifusion-based culture of brain slices [25], localized immunohistochemistry [26], and imaging mass spectrometry [27]. The open-space microfluidics system here is the Pixelated Chemical Display (PCD), which has been used for various processes over flat 2D surfaces, such as immunoassays [28]. A PCD comprises a blunt tip with multiple apertures and is installed in close vicinity of an immersed substrate. During its operation over the substrate, fluid streams are expelled through the injection apertures and re-collected through the aspiration apertures. As a result of the convective recirculation, fluid streams leaving a PCD form well-defined patterns over the substrate without mixing [29]. 4 Isolated flow sub-units which we refer to as “fluidic pixels” are created when a fluid stream injected above the surface is confined by neighbouring identical fluid streams, forming a repeating flow unit [30]. By modulating the design of the PCDs and the injection and aspiration flow rates, different sizes, numbers, and patterns of fluidic pixels can be achieved. Besides, the reagent content of fluidic pixels can be modulated over time with specific frequencies. Here, for the first time, we have utilized a PCD that creates nine square-shaped fluidic pixels for multiplexed reagent screening over 3D tumour models. To this end, we investigated the stability of the PCD when working over a large number of 3D biological samples deposited in a custom-built microwells array. To demonstrate the applicability of the platform across a whole spectrum of 3D tumour models, we perform a first experiment using the simplest 3D tumour models, spheroids, and later with the most complex 3D models, ex vivo tumour explants. We adapt our previously published paraffin-embedding lithography to transfer all samples simultaneously to the optimal cutting temperature (OCT) compound block while preserving their spatial orientation. Finally, we investigate the feasibility of using this method to study signalling pathways and cell fate in microdissected tumour tissues (MDTs).

## 2. Materials and Methods

### 2.1. Design and Fabrication of Parts

**Figure 1:**
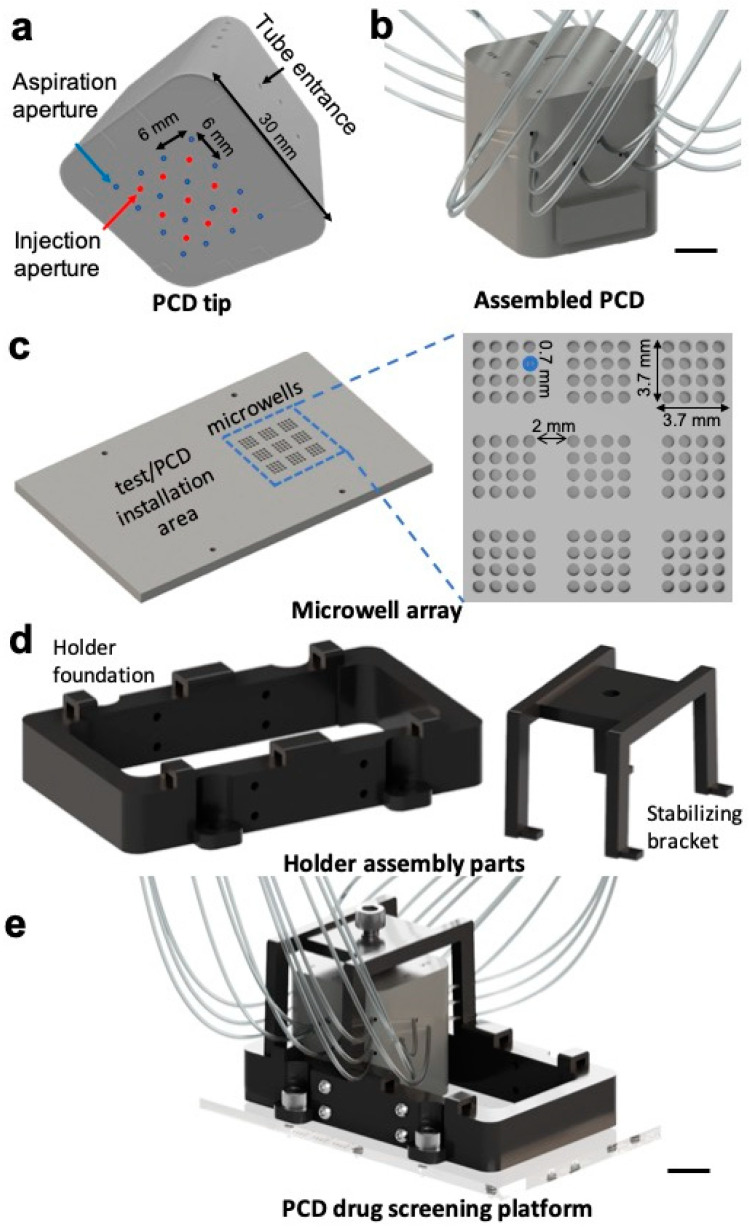
CAD figures of the drug screening platform parts created using Fusion 360 software: (**a**) a 3D printed nine-pixel PCD tip with injection and aspiration apertures. Each square-shaped pixel consists of an injection aperture in the centre of the square and four aspiration apertures on the corners; (**b**) fully assembled PCD showing the tubes connected to the PCD; (**c**) micromachined microwell array featuring 144 microwells to accommodate the tumour models; (**d**) 3D printed holder assembly parts with a holder foundation (left) that is attached to the microwell array slab and a bracket to stabilize the PCD once installed over the microwells (right), and (**e**) schematic of the fully assembled PCD drug screening platform. Scale bars = 1 cm.

We followed our previously published methodology to design and fabricate PCDs and manifolds that were used to branch the tubes [30]. PCDs and manifolds were designed using script-assisted CAD in Catia V5 (Dassault Systèmes, Vélizy-Villacoublay, France) and 3D printed using a stereolithography 3D printer (PICO2 HD, Asiga, Alexandria, Australia), as previously stated [28]. The resin used for the 3D printing was Pro3dure GR-1 black (P3GR1BLK-1L, Pro3dure medical GmbH, Iserlohn, Germany). After the printing, the excess resin was cleaned by sonication of the parts in an isopropanol bath, and then post-cured by UV exposure (Flash UV Curing Chamber, Asiga). To assemble the PCD (Figure 1a), 1/16” (RK-06419-01, Masterflex Tygon, Cole-Parmer, Vernon Hills, IL, USA) and 1/32” (RK-06420-01, Masterflex Tygon, Cole-Parmer) tubing was plugged and glued using a UV-sensitive resin (Figure 1b). Polycarbonate three-way stopcock valves (RK-30600-02, Cole-Parmer) were installed on the fluidic lines to enable switching between fluidic lines (Supplementary Figure S1). We designed a microwell array to accommodate the tumour models during the experiments. The microwell array was designed in Fusion 360 (Autodesk Inc., San Rafael, CA, USA) software, and micromachined using an MDX-40A milling machine (Roland DGA, Irvine, CA, USA) on a 1/8” PMMA slab (8560K239, McMaster-Carr, Elmhurst, IL, USA) (Figure 1c). The PMMA slab also featured a flat surface on the side of the microwell array to safely install the PCD and test its operation prior to biological experiments over tumour models (Figure 1c). We also designed and fabricated holder assembly parts (Figure 1d), including a holder foundation and a bracket to securely hold the PCD and the PMMA slab together, to stabilize the PCD and ensure its alignment over the microwell array. The holder assembly parts were designed in Fusion 360 (Autodesk Inc.) and 3D printed following the same protocol used to fabricate PCDs and manifolds. The holder foundation was fixed over the PMMA slab (Figure 1e) using screws and glue. Polydimethylsiloxane (PDMS; Sylgard^®^ 184 silicone elastomer kit, Dow Corning, Midland, MI, USA) was used to seal the holder foundation-PMMA slab interface and form a liquid-tight environment inside the holder foundation. Flow rates were controlled using AF1 microfluidic pressure pumps and microfluidic flow sensors (MSF-4, Elveflow, Paris, France). Microfluidic chips for drug testing on MDTs were fabricated using our previously published protocol [18].

### 2.2. System Operation

The operation of the system was controlled using a custom Python code (Python software foundation). To prepare the system, isopropanol was first streamed at 1 µL/s per aperture in all of the injection and aspiration tubes for at least 15 min to wet, prime, and sterilize the fluidic lines. The system was further infused for 5 min with PBS 1X (PBS 10X; 3072318, Wisent Inc., Saint-Bruno-de-Montarville, Canada) in all lines to purge isopropanol. Next, while still injecting PBS 1X in all of the injection and aspiration apertures, the PCD was installed inside the holder foundation, over the PMMA slab immersed in PBS 1X (Figure 1e). The PCD was first introduced over the flat surface next to the microwell array (i.e., annotated as “test/PCD installation area” in Figure 1c). Next, the experimental flow rates for injection and aspiration were administered, and the pixel formation was observed. An injection flow rate of 0.5 µL/s per injection aperture was used for all reagent streaming over live tissue. The aspiration to injection flowrate ratio was kept at 1.4. Then, the PCD was gently slid over the microwell array and fixated using the stabilizing bracket (Figure 1d,e). PBS 1X for formalin-fixed tissue, or neutral culture medium for live tissue, was administered for 20 min to rinse the microwells. Next, the reagents of interest were put in place. For formalin-fixed tissue staining experiments, Sytox^™^ Green (S7020, Thermo Fisher Scientific, Waltham, MA, USA), Nuclear Mask Red (H10326, Thermo Fisher Scientific), and DAPI (D1306, Thermo Fisher Scientific) were used. 10 mM solution of DAPI in PBS 1X was prepared and aliquoted. Dye solutions were diluted at a 1:500 ratio in PBS 1X. For live tissue staining experiments, Celltracker^™^ Green (CMFDA; C2925, Thermo Fisher Scientific), Celltracker™ Red (CMRA; C34551, Thermo Fisher Scientific), and a 1:1 solution of DAPI and Hoechst (Hoechst 33342, H3570, Thermo Fisher Scientific) were used. Dye solutions were diluted at a 1:500 ratio in the culture medium. At the end of the experiments, PBS 1X was injected for at least 10 min to rinse the fluidic lines and tumour models from the cellular dyes. Experiments were performed on a microscope stage and at room temperature. Injection reagent flasks were put in a water bath at 40 °C. Heating the reagent helps to prevent bubble formation in the fluidic lines and over the microwell array.

### 2.3. Finite Element Methodology

**Figure 2:**
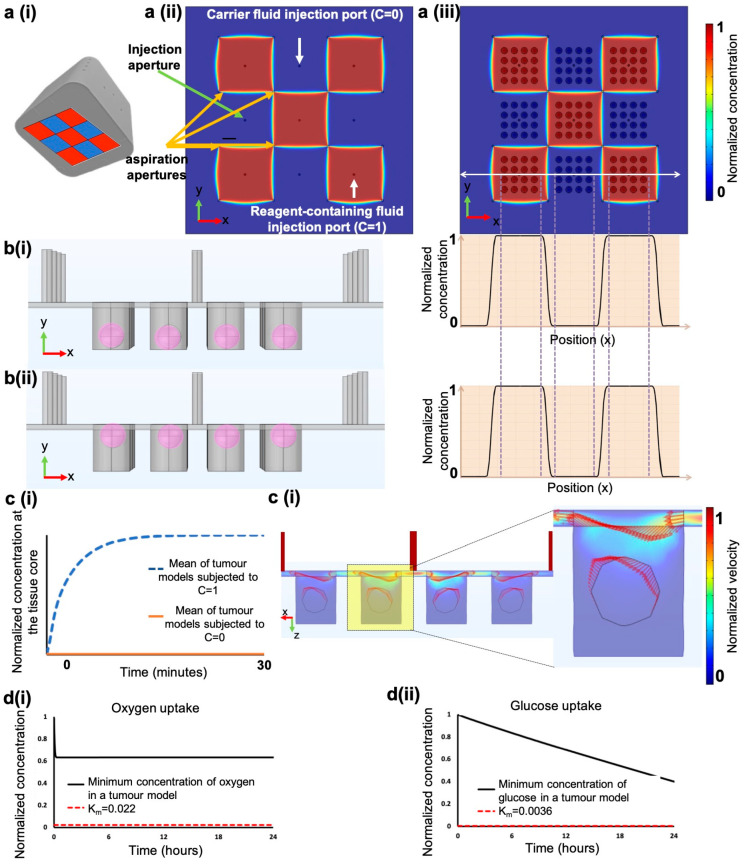
Numerical simulations of the PCD operation over tumour models. (**a**(**i**)) a schematic of the PCD and fluidic pixels modelled in the simulation, fluidic pixels formed over a flat surface (**a**(**ii**)) are comparable to those formed over the microwell array (**a**(**iii**)). (**b**) The positioning of the MDTs or spheroids in the microwells does not impact the operation of the PCD. (**c**(**i**)) Time required to reach a constant concentration throughout the tissue; (**c**(**ii**)) Arrow plots to visualize the distribution of the velocity field in the numerical model suggest the lack of free flow inside tumour models. (**d**(**i**)) Oxygen and (**d**(**ii**)) glucose consumption profile over time in tumour models cultured in the microwell array without perifusion. Tissues of up to 500 µm in diameter can survive in microwells for over 24 h, as the oxygen and glucose concentration stays above typical K_m_ values for cancer cells.

We used COMSOL Multiphysics© software v.5.6 (COMSOL Inc., Burlington, MA, USA) to simulate the convection and diffusion of reagents under the PCD and within tissue models. Passive diffusion of oxygen and glucose in the static culture of tumour models in between the medium changes was also modelled for the spheroid formation assay. The geometry of the model was drawn using built-in COMSOL drawing tools. The dimensions of the model can be found in Supplementary Table S1. All simulations were conducted at a constant biological temperature (37 °C). We used a time-dependent solver to model the PCD tumour model system and the spheroid formation assay. For the PCD tumour model system, Fick’s second law of diffusion and Navier–Stokes equation for laminar flow were applied using the “transport of diluted species in porous medium” module. In the simulations, the injection apertures were considered as inflows (i.e., source), injecting reagents with two interchangeably varying concentrations (i.e., reagent-containing fluid at C = 1 or carrier fluid at C = 0): starting from the top right side of the PCD tip, every other pixel received the reagent-containing fluid, resulting in a chequer-board pattern where 5 pixels were streaming the reagent-containing fluid solution and 4 pixels were streaming the carrier fluid (Figure 2a(i–iii)). The aspiration apertures were considered outflows (i.e., sinks). The aspiration to injection flow rate ratio was optimized to yield sharp pixels at the experimental flow rates and was kept constant throughout the simulation. The operational parameters of the model can be found in Supplementary Table S2. All the liquid compartments of the model had the physical properties (i.e., density and viscosity) of water at 37 °C. The porosity and hydraulic permeability are extremely low for the tumour model compartment [31,32]. With this in mind, we assumed the tumour models non-porous and used the diffusion coefficient of glucose in water to model the transport of reagents in the tissue models. For the spheroid viability assay, the “transport of diluted species module” was used to model the passive uptake of glucose and oxygen by tumour models. We first simulated oxygen transfer within the tumour models in the spheroid formation assay. We considered a constant oxygen concentration at the medium-air interface over the microwell array. PMMA is not gas-permeable, thus we imposed no-flux (Neumann) boundary conditions at the bottom and walls of the microwells. For glucose, we assumed continuity boundary conditions at the medium-tumour model interface. We used Michaelis–Menten (MM) kinetics to model cancer cells’ glucose and oxygen consumption rates in the spheroid formation assay. The average Michaelis–Menten uptake kinetics found in the literature [33,34,35] imply high consumption rates in the abundance of nutrients and decreased consumption rates when nutrients are depleted. The Michaelis–Menten constants refer to concentration thresholds, below which the normal cell metabolism is impacted [36]. We evaluated the minimum concentration of oxygen and glucose in the core of tissues of 500 µm in diameter. Tissue uptake and diffusion parameters are provided in Supplementary Table S2.

### 2.4. Cancer Cell Lines Xenograft Tumour Production

A human carcinoma cell line derived from an ovarian cancer tumour TOV21G (RRID: CVCL_3613) was used to produce mouse xenografts. Ovarian cancer cells were grown as monolayers (2D culture) in OSE medium (316-030-CL, Wisent Inc.) supplemented with 10% fetal bovine serum (FBS; Gibco™, Thermo Fisher Scientific), 55 mg/L gentamicin (Gibco™, Thermo Fisher Scientific), and 0.6 mg/L amphotericin B (Gibco™, Thermo Fisher Scientific). After reaching confluency, cells were detached with 0.25% trypsin-EDTA solution (Life Technologies, Carlsbad, CA, USA), and cell suspensions (1 000 000 cells) were mixed with Matrigel (BD Biosciences, Franklin Lakes, NJ, USA) at a 1:1 ratio and subcutaneously injected into the flank of immunodeficient NOD.Cg-Rag1tm1Mom Il2rgtm1Wjl/SzJ female mice (Charles River, Wilmington, MA, USA). Xenograft tumours were harvested once they reached a volume between 1500 and 2000 mm^3^. All animal procedures were performed in accordance with the Guidelines for the Care and Use of Laboratory Animals of the CRCHUM and approved by the Animal Ethics Committee (the Comité Institutionnel de Protection des Animaux).

### 2.5. MDT Production from Cell Line Xenograft Tumours

We used our previously published method [19,30] for the production of MDTs. Briefly, a tissue chopper (McIlwain, Ted Pella, Redding, CA, USA) was used to cut the xenograft into 350 µm-thick tissue slices. Tissue slices were kept in Hank’s Balanced Saline Solution (HBSS, 311-516-CL, Wisent Inc.) supplemented with serum and antibiotics. Tissue slices were further punched into MDTs using a 500 µm diameter tissue punch (Zivic Instruments, Pittsburgh, PA, USA) and kept in HBSS supplemented with antibiotics.

### 2.6. Microwell Preparation and MDT Loading in the Microwell Array

We designed and micromachined a microwell array to keep the tumour models in place at the PCD interface during the experiment. The microwell array features 9 groups of 16 microwells for a total of 144 microwells on a polymethylmethacrylate (PMMA) slab (Figure 1c). Each microwell is a cylinder of 700 µm in diameter and 900 µm in depth. Similar to PDMS devices [33], the microwell arrays were wetted and rendered hydrophilic by plasma treatment and rinsed with 100% ethanol. They were then sterilized by soaking in 70% ethanol for 15 min and prepared by incubation with a triblock copolymer (Pluronic^®^ F-108, Sigma-Aldrich, St. Louis, MO, USA) overnight (at least 16 h) at 37 °C in a 5% CO_2_ incubator. The microwell arrays were then rinsed with PBS 1X three times to purge the Pluronic^®^ F-108 solution. We adapted the previously published method of our laboratory to load the MDTs in microwells [18,19]. Briefly, the overlay liquid over the microwells was removed. 16 MDTs were picked using a 20 µL pipette and emptied over a microwell group. MDTs were diverted towards empty microwells using the pipette tip where they would fall into the microwells. In the case of more than one MDT falling in a microwell, the extra MDTs were pipetted out of the well and transferred to empty wells. This process was repeated for all 9 microwell groups.

### 2.7. Spheroid Formation Assay

The microwell array was prepared similarly for spheroids and MDTs. For spheroid culture, we drew a square of about 2 × 2 cm^2^ around the microwell array using a hydrophobic barrier pap pen (DAKO pen, Agilent, S200232) following the Pluronic treatment. This square provided a thin hydrophobic barrier that served to hold the culture medium and cell suspension within the microwell array area and avoided spillage over the PMMA slab. The microwell array was re-sterilized with ethanol 70% and rinsed with PBS 1X to purge ethanol. For spheroid formation experiments, we used a human squamous cell carcinoma FaDu (RRID: CVCL_1218) and a human colon cancer cell line HCT-116 (RRID: CVCL_0291). Cells were grown as monolayers (2D culture) in Dulbecco’s modified Eagle medium (DMEM; 11965118, Gibco™, Thermo Fisher Scientific) supplemented with serum and antibiotics. After reaching confluency, cells were detached and cell suspensions of 2,000,000 cells in 1 mL of culture medium were prepared. 400 µL of cell suspension was seeded over the microwell array, and the cell suspension was replenished three times to exchange the PBS 1X in the microwells with the cell suspension. We did not move the microwell array for 15 min following the cell seeding to let the cells sediment in the microwells. Then, the cell suspension over the microwell array was removed by gently drawing 400 µL of the cell suspension and adding 400 µL of medium to remove the floating cells over the microwells. The medium was changed every 24 h. To do so, the old culture medium was removed and 400 µL of fresh medium was added. The process was repeated three times to ensure the culture medium is replenished. Cell suspension and culture medium were added near one corner of the hydrophobic square around the microwell array and removed from the opposite corner.

### 2.8. OCT Embedding Protocol

Following fresh tissue experiments, MDTs underwent formalin fixation in the microwell arrays. 400 µL of formalin was added near one corner of the microwell array and removed from the opposite corner and repeated three times. The tumour models were incubated in formalin for 40 min and formalin was rinsed by three washes with PBS 1X. We then embedded the tumour models in agarose (Ultrapure™ Low Melting Point Agarose; 16520100, Thermo Fisher Scientific) to be able to transfer them directly from the microwell array to the OCT. For agarose embedding, an 8% solution of agarose in PBS 1% was prepared by dissolving 8 g of agarose in 100 mL PBS 1X and microwaving the solution for 80 s (4 cycles of 20 s) or until agarose powder was completely dissolved. The agarose in PBS solution was then cooled down to 62 °C. 400 µL of agarose solution was discharged and removed three times over the microwell array using a positive displacement pipette. The microwell array was placed in an oven at 60 °C for 30 min to ensure agarose permeates in the microwells and tissues. The microwell arrays were further cooled at 4 °C for 30 min, and the agarose layer was peeled off gently. If MDTs were left in the microwells after the removal of the agarose, a needle was used to remove them from the wells and add them to the agarose tissue array. The agarose block was cut to separate the microwell groups, and microwells subjected to the same treatment condition were placed in the same plastic moulds, ensuring that tissues were touching the bottom of the plastic mould. OCT was poured gently over the agarose block to prevent bubble formation. Plastic moulds were placed on a flat and levelled surface in dry ice and cooled down for 20 min for OCT to solidify. Each OCT block was sliced into 5 µm-thick sections using a cryostat, and each section was placed on a TOMO^®^ hydrophilic adhesion slide (Matsunami, Bellingham, WA, USA).

### 2.9. Tumour Model Treatment with TNF

For cytokine stimulation experiments, MDTs were exposed to a neutral culture medium, or to 20 ng/mL of TNF solution (Recombinant TNF alpha human; 300-01A, PeproTech, Thermo Fisher Scientific) in culture medium for either 30 min or 240 min. These time points were selected based on a previous similar experiment by our laboratory [19]. The TNF treatment using the PCD lasted 300 min. First, the PCD streamed a neutral culture medium at every pixel for 20 min. Then, we streamed TNF in one group (3 pixels) while the two remaining groups (6 pixels) received a neutral culture medium. At 230 min, TNF streaming was started in a second group. For the next 50 min, TNF was streaming at 6 pixels, all the while the control group on the same 3 pixels received culture medium. At 280 min, we swapped all reagent flasks for PBS 1X, and PBS 1X was streamed for 20 min to rinse the tumour models. The PCD was then removed, and the immersion liquid over the microwell array was withdrawn. Tumour models underwent OCT embedding for further histopathology processes. We followed our group’s protocol for MDT treatment on-chip [19]. For the 2D culture of cells, cells were treated with TNF for 5 min or 2 h.

### 2.10. Histopathological Staining

OCT sections underwent immunofluorescent (IF) staining to assess the expression of p65 protein (Anti-NFkB p65 protein; SC-8008, Santa Cruz, TX, USA) and DAPI in the tumour models. IF staining was performed using the BenchMark XT automated stainer (Ventana Medical System Inc., Tucson, AZ, USA). Antigen retrieval was carried out with Cell Conditioning 1 (#950-123, Ventana Medical System Inc.) for 90 min for primary antibodies. Mouse anti-p65 (1:200) antibody was automatically dispensed. The slides were incubated at 37 °C for 60 min and secondary antibodies were incubated at room temperature on the bench. We used our laboratory’s protocol to quantify the TNF response in 2D culture [37]. Briefly, cells were seeded onto coverslips at 20,000 cells/well in 24-well plates. After 24 h, cells were incubated with TNF solution for 5 min or 2 h. Cells were fixed with formalin for 15 minutes at room temperature, washed using PBS 1X, permeabilized with 0.25% Triton (Triton™ X-100 solution; 93443, Sigma-Aldrich), and incubated with mouse anti-p65 (1:400) overnight. The primary antibody was detected by incubation with a secondary antibody for 60 min. Coverslips were mounted onto slides using Prolong Gold^®^ anti-fade reagent with DAPI (14209 S, Life Technologies Inc.). All sections were scanned with a 20×/0.75 NA objective with a resolution of 0.3225 μm (bx61vs, Olympus, Toronto, ON, Canada).

### 2.11. Quantification of Immunofluorescent Staining

To measure the fluorescence intensity (FI) of tumour models stained using the PCD, an open-source image processing software (Fiji) was used [38]. 3 spheroids were randomly selected in each fluidic pixel, and the corrected FI per area (subtracting the background FI from tissue FI) was calculated for each fluorescent channel. The average corrected FI per area of the 3 pixels subjected to the same treatment was compared between the 2- and 3-h incubation time for each channel. To quantify protein expressions using immunofluorescent staining, we used VisiomorphDP software (VisioPharm, Hørsholm, Denmark) [39,40]. Briefly, the tissue core surface area was detected through the DAPI channel. The nuclear signal of p65 was quantified by dividing the surface area of p65-positive nuclei by the total surface area of the nuclei. We used a similar approach for quantifying p65 translocation in the 2D culture of cells [39].

### 2.12. Statistical Analysis

Statistical analyses were performed in GraphPad Prism version 8.0 (San Diego, CA, USA) using the non-parametric one-way ANOVA Kruskal–Wallis test and post hoc Dunn’s test. For the TNF treatment experiment, a minimum of 3 MDTs per condition per experiment were analyzed, and experiments were repeated three times (N = 3). Unless otherwise stated, all data are reported as the mean ± standard error of the mean (SEM). The reported *p*-values were generated using a post hoc test (Dunn’s test).

## 3. Results

### 3.1. Design and Fabrication of the Pixelated Chemical Display Drug Screening Platform

The design of PCD is highly modular, and massively parallel PCDs can be built [30]. Our group has previously demonstrated theoretically and experimentally the operation of up to 144 fluidic pixels (12 × 12) and demonstrated that the number of active pixels and their reagent content can be modulated without altering the stability of the system [40]. Based on our previous findings, we adapted a 9-pixel PCD for tissue culture and drug screening. Each pixel is 36 mm^2^ (6 × 6 mm^2^) such that the resulting array fits within a paraffin cassette for later embedding. For this work, each group of 3 pixels was connected to the same reagent flask to create experimental triplicates. Three different conditions were tested in each experiment. The microwell array features 9 groups of 16 microwells corresponding to the pixels of the PCD (Figure 1c). Each microwell group is covered by an independent fluidic pixel when the PCD is aligned over the microwell array.

### 3.2. Perifusion vs. Perfusion

In designing culture systems for 3D biology, it is important to differentiate between perfusion- and perifusion-based devices. Perifusion-based microfluidic devices have been developed to preserve the viability of larger tissue explants over a longer time [41,42,43]. Tumour tissues are dense structures with permeabilities that are orders of magnitude below the permeability of flow channels [31,32]. Creating convective flow inside tumour models (i.e., perfusion) is not feasible unless flow around them is prevented. In most cases, when samples are small (<1 mm), perifusion is sufficient to avoid any form of starvation, anoxia, and necrosis in tissue [35]. Perifusion-based devices, while presenting a technical breakthrough, have extremely limited throughputs [44,45].

### 3.3. Pressure Pump-Operated Fluidic Lines

Syringe pumps were previously used to operate PCDs [28,30] as they offer precise and simple control, and are commonly used in microfluidic systems [46]. However, syringe pumps often have relatively high minimum working flow rates. Higher precision syringe pumps require frequent recharging of reagents in syringes [47]. To avoid these limitations, we used pressure pumps in this work. Pressure pumps enable pressurizing of a wide range of flask sizes (from microliters to litres capacity) and thus allow for longer-run experiments. More importantly, a single pressure pump can be used to pressurize several reagent flasks, whereas each syringe pump is dedicated to a single syringe. This will also greatly reduce costs. Different flow rates can be achieved in different fluidic lines pressurized by one pump by controlling the hydraulic resistance of the tubes (i.e., by using different sizes of tubing). In this work, two pressure pumps were used to operate the PCD: one for the injection groups and one for the aspiration. Similar to our previous works, we used 3D-printed manifolds to deliver fluids from one pump into all the pixels sharing the same reagents [30]. Tubes connecting the manifolds to the PCD were used as precision hydraulic resistors to match the flow rate from all apertures. Four precision flowmeters were installed on the fluidic lines to measure the flow rate for the four injection and aspiration groups. A closed-loop control system with a feedback loop control (a.k.a., proportional–integral (PI) controller) was developed to control the pressure-driven flows. The PI controller estimates the deviation between the target and the measured injection flow rates and regulates the pressure to reduce deviations in real time. Moreover, we added three-way stopcock valves on the fluidic lines to enable on-demand reagent switching between the various reagent flasks (e.g., priming reagents such as ethanol and isopropanol, culture medium, and biochemicals reagents). Switch valves enable us to add or remove reagent flasks without interrupting the system (Supplementary Figure S1). We refer to the PCD, microwell array, pumps, and fluidic lines complex as the PCD drug screening platform.

### 3.4. Finite Element Simulations

Our group has previously studied the mass transport, stability, and reconfigurability of PCDs using 2D convection-diffusion finite-element methods and has demonstrated that stochastic errors such as minor pressure, flowrate changes, or clogging of one aperture do not impact the PCD’s operation [30]. Here, we conducted numerical simulations to gain insight into the quality, crosstalk, and stability of fluidic pixels when the PCD is working over 3D structures, such as tumour models. We added a secondary set of simulations to predict the convective-diffusive transport of diluted reagents in microwells and inside tumour models. We used experimental geometrical and operational parameters: flow rates were selected based on the minimum flow rate that we could achieve with the pressure pumps to have sharp and stable pixels while minimizing reagent consumption. To visualize the pixel formation and crosstalk, we modelled a PCD that functioned in a checkerboard pattern: five injection apertures inject a reagent-containing fluid (i.e., C = 1), and four injection apertures inject a carrier fluid (i.e., C = 0) (Figure 2a(i–iii)). Simulation results suggest that the presence of microwells and tumour models does not disturb the pixels’ shapes similarly to working over flat impermeable surfaces (Figure 2a(ii)): stable crosstalk-free pixels are formed over the microwell array (Figure 2a(iii)). Moreover, we investigated the impact of the tumour model positioning in microwells on fluidic pixels. We modelled a scenario in which random tumour models were partially sticking out of the wells and touched the PCD. We observed that, regardless of the tumour model positioning in the well, the pixels are stable (Figure 2b). We also measured the amount of time required to reach a constant concentration of an injected reagent inside the tumour models with zero initial concentration. Simulation results predict that the system reaches a steady state in less than 20 min (Figure 2c(i)). The time to reach this state was set as the transition time in the experiments; upon the change of a reagent flask, reagents were streamed for 20 min before starting the experiment countdown. We then evaluated the shear stress induced on the cells by the flow and observed that the maximum shear stress imposed by the PCD is 0.0021 Pa, which is approximately 500 times less than the physiologically safe shear stress regime for sensitive cells (~1 Pa) [48]. By visualizing velocity fields, we observed that there is no convective flow inside the tumour models (Figure 2c(ii)), showing the diffusion dominant transfer of reagents inside tumour models. Overall, our simulations predict that the PCD provides excellent control over the fluidic pixels, and locally perifuses the tumour models. Finally, to model the spheroid formation assay (i.e., the static culture of 500 µm-tumour models in the microwell array), we used passive diffusion and Michaelis–Menten kinetics parameters for oxygen and glucose consumption by cancer cell lines [35]. The numerical model predicts that spheroids have access to sufficient levels of oxygen and glucose over 24 h (i.e., the typical medium refreshment interval) (Figure 2d).

### 3.5. High-Throughput Formation of Cancer Cell Line Spheroids Is Possible in the Microwell Array

**Figure 3:**
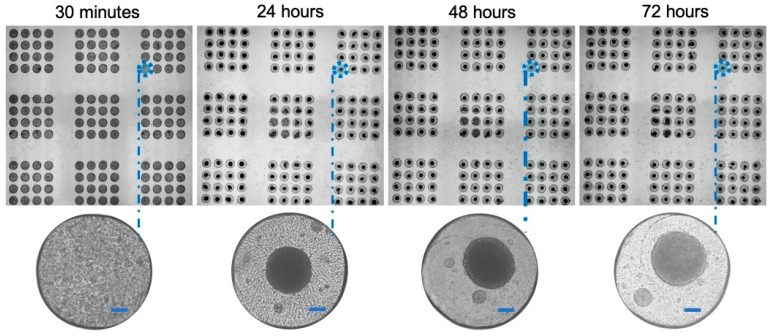
Formation of uniform and compact spheroids of colon cancer cell line HCT-116 in the microwell array over time. Scale bars = 100 µm.

Cancer cell line spheroids are self-formed spherical cell aggregates formed from one or more cancer cell lines [49]. Spheroids are the simplest and most often used 3D tumour models. The commonly used technique to form spheroids is to seed a high-density suspension of cells on a non-adherent surface. Spheroids will form if the cell–cell adhesion forces are greater than the cell-surface adhesion forces [50]. To meet our claim about the amenability of PCD to work with different tumour models, we optimized the surface modification technique and cell seeding densities based on previous findings to form spheroids directly in the microwell array [51,52,53]. We further formed spheroids from squamous cell carcinoma and colorectal cancer cell lines to test the practicality of the approach. Figure 3 and Supplementary Figure S2 show that we can form uniform spheroids in the microwell array in 48 h. Spheroids were later subjected to the PCD for dynamic cellular staining.

### 3.6. The PCD Drug Screening Platform Enables Dynamic Multiplexed Staining of Spheroids

**Figure 4:**
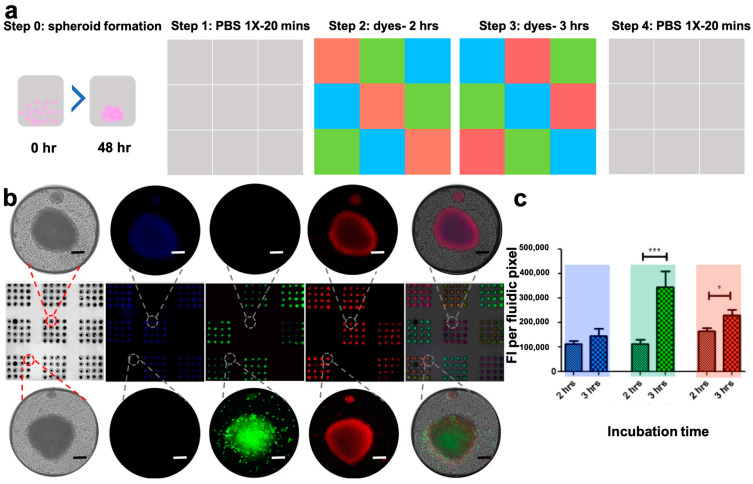
Crosstalk-free multiplexed staining of tumour models using the PCD. The PCD is used to stain HCT-116 spheroids 48 h after cell seeding. Spheroids were first subjected to three different cellular dyes streaming at the nine pixels of the PCD for two hours. Then, the reagents were switched to stream a different dye at each pixel for 3 h. Spheroids were imaged using an inverted fluorescent microscope after rinsing out the dyes. Staining protocol (**a**), micrographs of stained spheroids taken at the end of the experiment (**b**), and quantification of Fluorescence Intensity (FI) of spheroids for each channel (**c**). Longer incubation with fluorophores results in a higher fluorescent emission from the spheroids. Blue: Hoechst and DAPI, green: Celltracker™ Green, red: Celltracker™ Red. Scale bars = 100 µm. (**b**,**c**) represent one assay. 3 Spheroids in each pixel (9 spheroids per channel) were used to produce the plot in (**c**). Error bars = SEM. * *p* < 0.05; *** *p* < 0.0001.

To test the performance, stability, and precision of the drug screening platform, we used it to stain spheroids formed in the microwell array. Two phases of reagent streaming over spheroids were used to test the stability of the PCD over subsequent changes of reagents, and to verify its potential for dynamic reagent screening. In this experiment, the PCD first streamed culture medium for 20 min over the microwell array containing spheroids. Subsequently and without interrupting the system, the culture medium was replaced by three cellular dyes that were streamed in the 9 pixels of the PCD for 2 h in a predetermined pattern. We then switched the reagent flasks, subjecting spheroids to a second dye. We used the same reagents (three cellular dyes) all throughout the experiment, but spheroids were exposed to a different colour on the second part of reagent streaming than they were exposed to for the first 2 h to create an alternate pattern. The second part of the reagent streaming went on for 3 h. There were no pauses or wash steps between the two reagent streaming parts. At the end of the incubation period, cellular dyes were swapped with PBS 1X to purge the dyes (Figure 4a). Spheroids were imaged at the end of the experiment and removal of the PCD, using fluorescence microscopy. Spheroids under each fluidic pixel had been exposed to two different fluorescent dyes. For each fluorescent channel, we had three fluidic pixels (3 × 16 microwells) representing 2 h of dye exposure and a further three pixels exposed to dye for 3 h. We did not observe staining of the spheroids with a dye they have not been exposed to in any of the pixels. These results show crosstalk-free staining of spheroids with the colours of interest (Figure 4b). We further assessed the FI per unit area of spheroids for different channels and demonstrated that spheroids subjected to a dye for 3 h have a higher FI than spheroids subjected to the same dye for 2 h (Figure 4c).

### 3.7. The PCD Drug Screening Platform Can Handle Fragile Ex Vivo Tumour Tissue Explants

Ex vivo tumour tissue explants provide an excellent tumour model since they are readily available from biopsy or surgery, do not require disintegration, and mirror the individual’s tumour features including the histological and gene expression profiles [19,54]. However, they are the least frequently used 3D tumour model due to the frailty of the tissue and limited throughput. Our group has devised a methodology in which tumour tissues are dissected into sub-millimetre-sized fragments and cultivated on microfluidic chips [33]. The MDT methodology yields many MDTs from small primary tissue and maximizes tissue viability [19]. Its applications are restricted by the fact that limitations are imposed due to its closed microfluidic chips. To free the system from these limitations, we investigated the application of the PCD drug screening platform to MDTs. Fresh or formalin-fixed MDTs produced from xenografted tumour tissue were deposited in the microwell array and subjected to the PCD. The PCD streamed cellular dyes over the MDT. Images of whole MDTs captured using the fluorescent microscope showed crosstalk-free staining of MDTs with the intended colours, similar to what was observed for spheroids (Supplementary Figure S3).

### 3.8. Tumour Tissue Microarray

**Figure 5:**
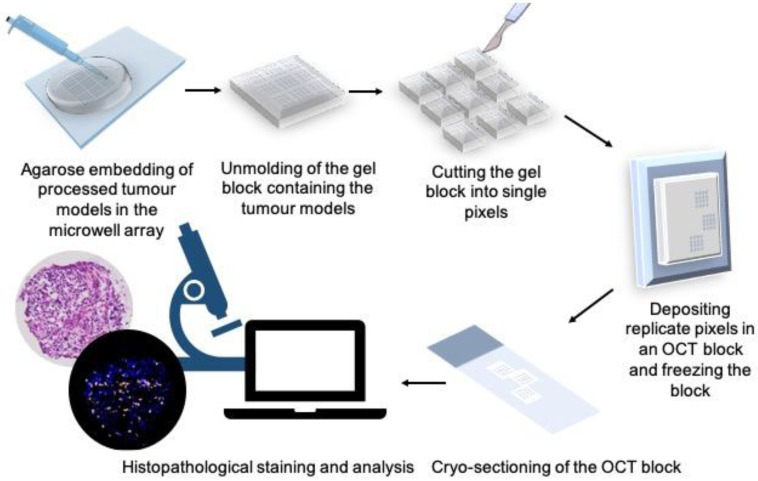
Protocol developed to remove the tumour models from microwells while preserving their address for further histopathology analyses. Tumour models are first embedded in agarose and the gel is removed from the microwell array. Microwell groups exposed to different fluidic pixels are separated, and tumour models that have been subjected to the same treatment condition are grouped in an OCT block. OCT blocks are sectioned in 5 µm sections to visualize the tissue core and for further immunostaining.

Fluorescence microscopy captured the mean fluorescence emission of tumour model structures, but did not allow us to examine the distribution of reagents throughout the tumour models. To demonstrate this capability in our system, we developed a methodology to take tumour models out of the microwells and directly embed them in a freezing medium. It is also essential to keep the arrangement and orientation of tumour models as they were in the microwells to be able to correlate them with the treatment conditions (in each fluidic pixel) that they have been exposed to. For this, we adapted a technique previously described by Jones and Calabresi [55] to first embed the tumour models in a hydrogel (agarose) in their microwells. Then, the hydrogel block containing the tumour models is de-moulded from the microwell array and re-embedded in the OCT compound. The OCT blocks were then sectioned into 5 µm-thick slices, and slices were used for further histopathological staining and analysis (Figure 5). It is noteworthy that this protocol makes the PCD drug screening platform compatible with standard histopathology practice. Supplementary Figure S3 shows cryosections of MDTs that were stained using the PCD for different durations and underwent the agarose and OCT embedding protocol.

### 3.9. The PCD Drug Screening Platform Enables the Tracking of Biological Responses in Tumour Models

**Figure 6:**
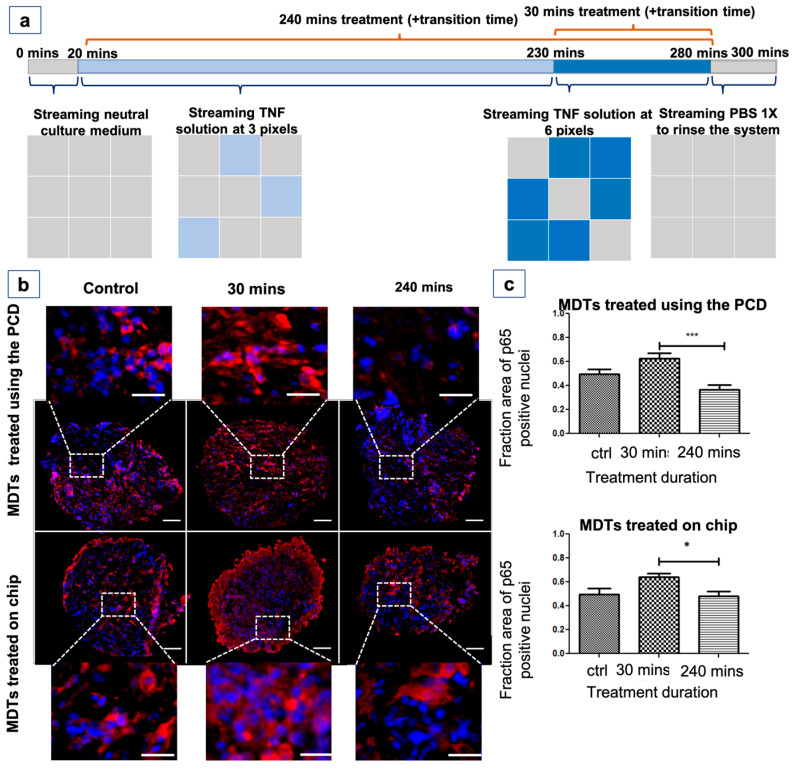
The PCD drug screening platform can recreate the on-chip response of tumour models to a cytokine. Xenograft cell line MDTs (TOV 21G) were treated with a TNF solution for different durations using the PCD, as well as on-chip for comparison (**a**). Fluorescence micrograph of cryosections of OCT-embedded tissue that have undergone immunofluorescent (IF) staining (**b**). Changes in the nuclear translocation of p65 were quantified in the IF staining on MDT cryosections (**c**). Red: p65 and blue: DAPI. Scale bars = 20 µm for zoomed-in MDTs, and 100 µm for whole MDTs. N = 3 experiments and a minimum of three MDTs tested per condition. Error bars = SEM. * *p* < 0.05; *** *p* < 0.0001.

After demonstrating that the PCD platform is capable of forming crosstalk-free fluidic pixels over various tumour models, we sought to examine the ability of the technology to follow various biological responses in tumour models. For this, we assessed the response of Nuclear factor kappa B (NF-κB) transcription factors in MDTs to a cytokine (tumour necrosis factor [TNF]) stimulation. The NF-κB transcription factor is reported to play a role in tumour angiogenesis and invasiveness and is a possible target to improve the clinical diagnosis and prognosis [56]. NF-κB resides in the cytoplasm of every cell and is translocated to the nucleus when activated by various stimuli such as cytokines, viruses, and free radicals [57]. TNF is a proinflammatory cytokine that is known to activate NF-κB [58]. Real-time monitoring of nuclear translocation of Nf-κB proteins in previous studies revealed a rapid increase in the nuclear signal of sub-units of NF-κB that peaks a few minutes after the exposure, followed by a decline in the nuclear signal of proteins [59,60]. With this in mind, and based on a previously established experimental protocol [19], we used the PCD to expose MDTs produced from cell line xenografts to TNF for 0, 30 min, or 240 min by progressively switching on TNF delivery in certain pixels by replacing neutral culture medium with a TNF solution (Figure 6a). We evaluated the nuclear signal of p65, an NF-κB subunit, by IF staining (Figure 6b). The quantification of the IF staining showed an increase in the nuclear signal of p65 in MDTs that have been subjected to TNF stimulus for 30 min compared to the control group. The p65 nuclear signal dropped in the MDTs that were treated for 240 min (Figure 6c). This is also expected since p65 translocation is known to be reversible [58]. To further validate the results, we performed parallel experiments on MDTs produced from the same xenograft that were cultured on chips. The on-chip MDT treatment experiment is a repeat of a protocol previously published by our laboratory for other cell-line xenografts MDTs [19]. Similar responses were also observed in MDTs on chips (Figure 6c). 2D cell cultures of the same cell line treated with TNF for 5 min and 2 h showed similar results (Supplementary Figure S4), substantiating the results seen in MDTs. The results showcase that the PCD drug screening platform can reflect the response of tumour samples to biological stimuli. It is noteworthy that we expected to see a more significant difference between the control and treatment groups. This can be due to the high baseline levels of nuclear p65 in the cell line selected [61]. We are also aware that the serum factors—such as growth factors, cytokines, soluble cytokine receptors, and macroglobulin—present in the serum-supplemented medium that were used may influence the TNF assessment [62]. It is also reported that the TNF-induced gene expression oscillates in time [63], hence the treatment durations might need to be individualized for each cell line and TNF assessment should be a subject of future investigation. Nevertheless, these preliminary results showcase that the PCD drug screening platform can yield similar on-chip responses to biological stimuli.

## 4. Discussion

The need to improve the predictive power of in vitro and ex vivo model systems to maximize the chances of success in clinical trials has made 3D tumour models, such as microdissected tissue and cancer cell line spheroids, attractive in preclinical settings [64,65]. Thus, it is essential to implement tools and techniques to automate drug screening on 3D tumour model systems and make them compatible with clinical practices. To address this, we introduced a drug screening platform for automated simultaneous streaming of up to 9 reagents on 144 tumour models. We used human cancer cell line xenograft and spheroid models to validate the potential of the PCD drug screening platform for multiplexed and dynamic streaming of biochemicals over tumour models. Microtissues processed in the drug screening platform can directly be transferred to an embedding medium and undergo various endpoint measurements (i.e., immunohistochemistry, immunofluorescence, and H&E). These measurements are standard protocols in clinical and pharmaceutical practices and allow the monitoring of multiple biological pathways. Furthermore, because the platform is amenable to different 3D tumour models, it allows the co-culture of spheroids, organoids, and ex vivo tumour tissue explants. In turn, this enables comparison of treatment efficacy on various tumour models. The main drawback of the PCD arises from the continuous streaming of reagents. Even though flow rates are extremely low, streaming over several hours consumes a considerable amount of reagents, and thus limits applications in cases where reagents are extremely expensive (e.g., recombinant protein drugs). However, a highly parallel drug screening assay using the PCD would probably even be worthwhile despite the high reagent consumption. We have reported a low number of large pixels (9 × 6 mm^2^) in this article. However, PCDs of up to 144 × 1 mm^2^ pixels have been produced routinely in our laboratory with successive reagent changes as fast as 1 change per 30 s. This makes the PCD drug screening platform appealing for highly parallel and dynamic assays. The reconfigurable sizes and numbers of pixels along with the fast reagent change will speed up the throughput.

Compared to traditional well plate-based approaches [66] and their downsized microfluidic counterparts such as InSphero GravityTRAP™ [67], idenTx™ [68], and Organoplate^®^ [69], our platform does not require the manual delivery of reagents to microtissues or the use of robotic liquid handlers. This should greatly reduce the time and cost required to perform the experiments. Moreover, the possibility of the direct transfer of tumour models to an embedding medium reduces the potential tissue damage and makes our platform more efficient compared to previous approaches where each individual sample is transferred from well plates to an embedding medium. While this work only focuses on short-term on-bench experiments, it provides robust evidence for the capacity of the PCDs to manipulate 3D tumour models. Next, we plan to utilize the platform for longer-term experiments inside a CO_2_ incubator. Experiments using primary human tumour tissues will also be a matter of future investigation.

## 5. Conclusions

To address the need for efficient preclinical drug screening assays, we have utilized an open-space microfluidic platform for multiplexed delivery of reagents to 3D tumour models. The proposed drug screening platform allows for testing different reagents on 144 tumour models in a single experiment and is amenable to various 3D tumour models such as spheroids or ex vivo tumour explants. Several proof-of-concept applications are demonstrated. The platform has the potential for higher throughput culture and analysis of 3D tumour models and will be of great benefit to the pharmaceutical drug development and personalized cancer medicine fields.

## Data Availability

Not applicable.

## Supplementary Materials

The following supporting information can be downloaded at: https://www.mdpi.com/article/10.3390/cancers15041060/s1, reference citation [35,70,71,72,73,74,75,76,77]. Figure S1: Fluidic connections in the PCD drug screening platform. The use of valves allows for switching between the streaming of various reagents and the flowrate sensors allow for control and validation that the platform is working correctly. OD; outside diameter; Figure S2: Spheroid formation in the microwell array using FaDu cell line. Scale bar = 2 mm; Figure S3: Representative micrographs of formalin-fixed MDTs produced from TOV21G cell line (a) brightfield image of MDTs deposited in the microwell array; MDTs stained with various cellular dyes using the PCD for (b) 2 h and (c) for 3 h. b(i) and c(i) were taken at the end of the staining experiment after the samples were rinsed with PBS 1X and the PCD was removed. Stained MDTs were removed from the microwells using the tissue microarray protocol and embedded in the OCT. b(ii) and c(ii) are representative images of cryosections of tissue cores of MDTs that have been stained for 2 and 3 h, respectively. Images taken from the tumour model cores that have been treated with cellular dyes for different amounts of time show that core cells are not stained in the shorter treatment durations. a, b, and c represent different assays. Scale bar = 100 µm for single MDTs and 2 mm for the microwell array. Figure S4: Time-dependent treatment of 2D culture of TOV21G cells with TNF shows a response similar to MDTs treated on-chip or using the PCD. This further validates the potential of the PCD drug screening platform to predict the response of 3D tumour models to stimuli. Scale bar = 20 µm, N = 3 experiments. Table S1: dimensions of the systems; Table S2: Tissue uptake parameters, diffusion properties, the PCD working condition.
