# Supplementary material for: Pixelated Microfluidics for Drug Screening on Tumour Spheroids and Ex Vivo Microdissected Tumour Explants
Source: Cancers (Basel). 2023 Feb 7;15(4):1060. doi: 10.3390/cancers15041060 (PMC9954565; doi:10.3390/cancers15041060)
Supplement: Supplementary file 1 [file cancers-15-01060-s001.zip › cancers-2139569-supplementary.docx]

Pixelated microfluidics for drug screening on tumour spheroids and *ex vivo* microdissected tumour explants

Dina Dorrigiv,^a,b^ Pierre-Alexandre Goyette,^b^ Amélie St-Georges-Robillard,^a,c^ Anne-Marie Mes-Masson,^a,d^ and Thomas Gervais^a,b,c^*

Supplementary figures


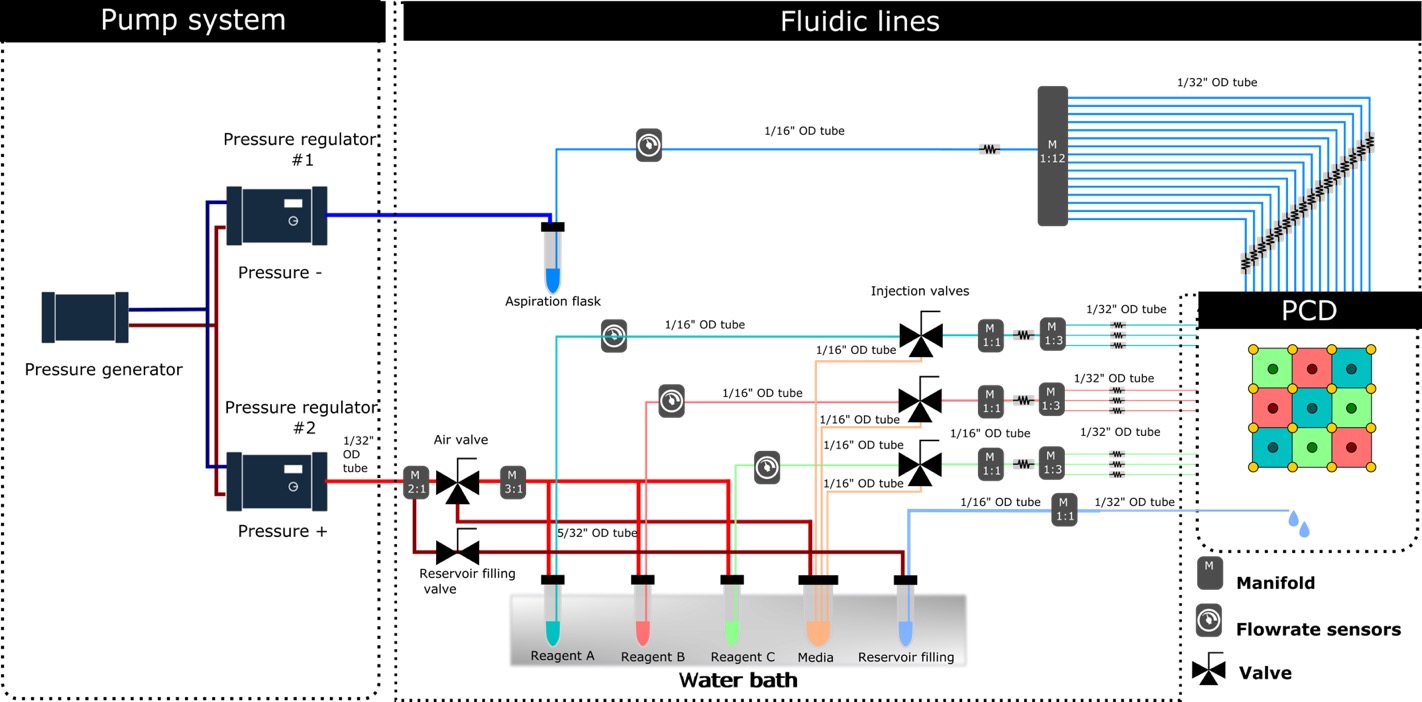


**Figure S1**. Fluidic connections in the PCD drug screening platform. The use of valves allows for switching between the streaming of various reagents and the flowrate sensors allow for control and validation that the platform is working correctly. OD; outside diameter.


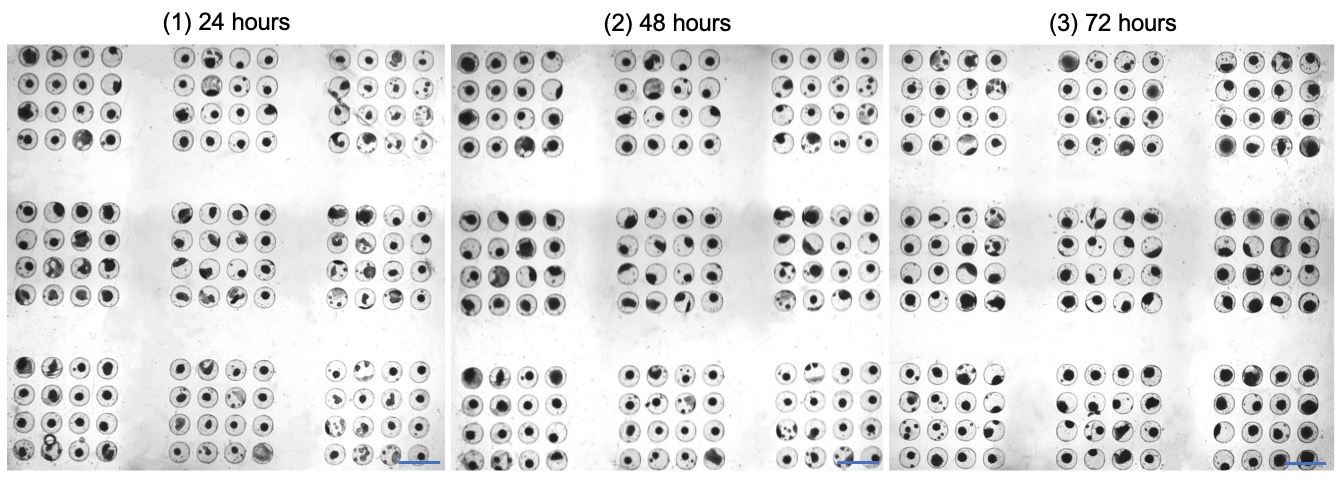


**Figure S2**. spheroid formation in the microwell array using FaDu cell line. Scale bar= 2 mm


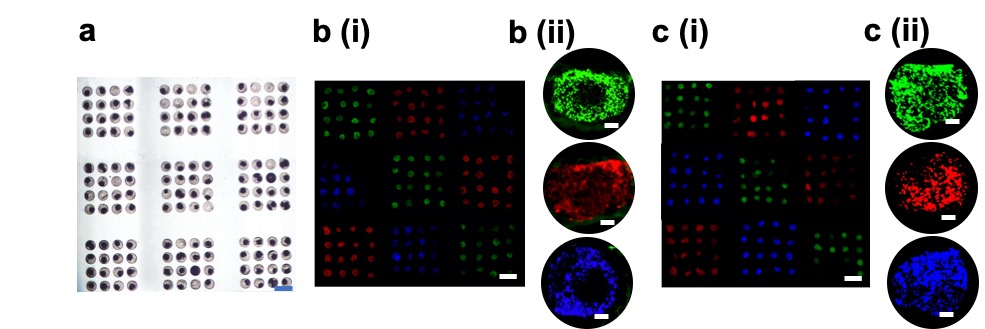


**Figure S3**. Representative micrographs of formalin-fixed MDTs produced from TOV21G cell line a) brightfield image of MDTs deposited in the microwell array; MDTs stained with various cellular dyes using the PCD for b) 2 hours and c) for 3 hours. b (i) and c (i) were taken at the end of the staining experiment after the samples were rinsed with PBS 1X and the PCD was removed. Stained MDTs were removed from the microwells using the tissue microarray protocol and embedded in the OCT. b (ii) and c (ii) are representative images of cryosections of tissue cores of MDTs that have been stained for 2 and 3 hours, respectively. Images taken from the tumour model cores that have been treated with cellular dyes for different amounts of time show that core cells are not stained in the shorter treatment durations. a, b, and c represent different assays. Scale bar= 100 µm for single MDTs and 2 mm for the microwell array.


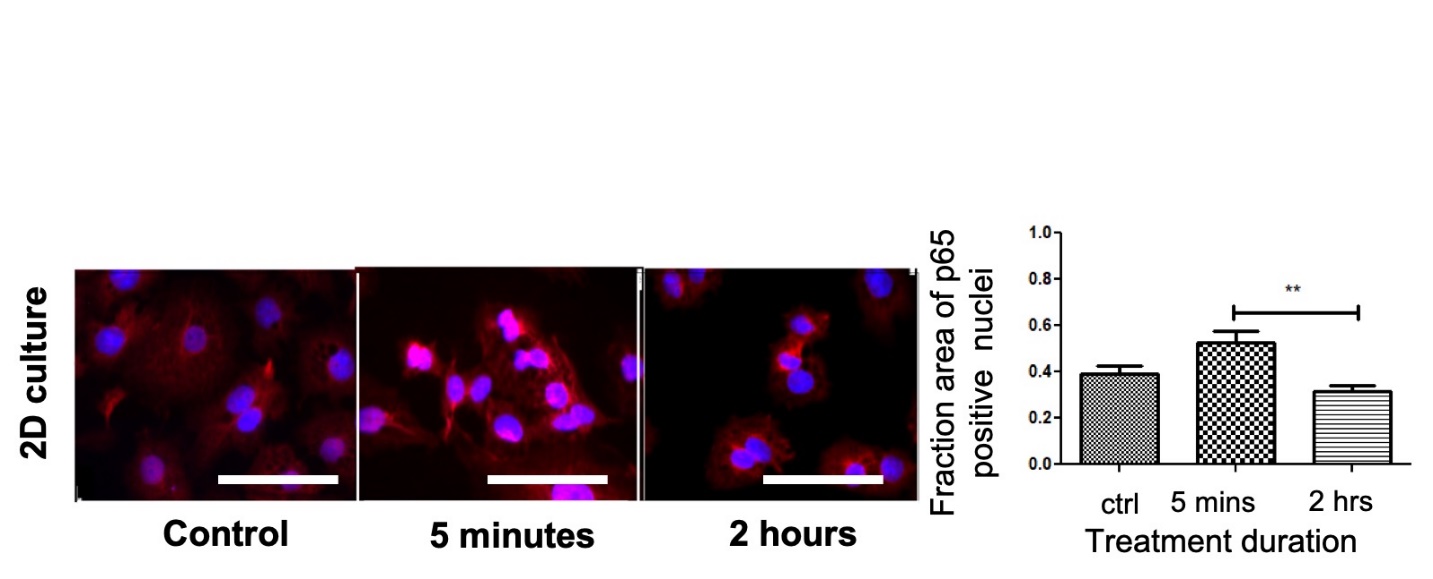


**Figure S4**. time-dependent treatment of 2D culture of TOV21G cells with TNF shows a response similar to MDTs treated on-chip or using the PCD. This further validates the potential of the PCD drug screening platform to predict the response of 3D tumour models to stimuli. Scale bar=20 µm, N=3 experiments. Error bars = SEM. * p < 0.05; ** p < 0.01; *** p < 0.0001; **** p < 0.00001

Supplementary tables

**Table S1.** dimensions of the systems

| **Parameter** | **Value (µm)** |
| --- | --- |
| **Microwell array** | |
| well height | 900 |
| well width | 700 |
| well length | 700 |
| Distance between microwells | 1000 |
| **PCD** | |
| Gap between the PCD and the microwell array | 100 |
| Aperture diameter | 200 |
| Distance between apertures (i.e., pixel size) | 5000 |
| Number of aspiration apertures | 16 |
| Number of injection apertures | 9 |
| **Tissue** | |
| Tissue diameter | 450 |

**Table S2**. tissue uptake parameters, diffusion properties, the PCD working condition

| **Parameter** | **value** | | |
| --- | --- | --- | --- |
| **Diffusion/Reaction Parameters** | | | |
| Diffusion constant of glucose (cm^2^/s) | Tissue | 2.7x10^-6 1,2^ | |
|  | Medium | 9.27x10^-5^  ^1^ | |
|  | Agar 5% | Same as water ^3,4^ | |
| Diffusion constant of oxygen (cm^2^/s) | Tissue | 1.8x10^-5^ | |
|  | Medium | 2.6x10^-5^ | |
|  | Agar 5% | 2x10^-5 4^ | |
|  | PDMS | 3.4x10^-5^  ^5,6^ | |
| Saturation concentration (mM) | Oxygen | Tissue | 1.02 |
|  |  | medium | 0.21 |
|  |  | Agar 5% | 0.21colagen^9^ |
|  |  | PDMS | 1.43 |
|  | Glucose | 11 | |
| Oxygen partition coefficient (relative solubility of oxygen) | PDMS-Medium | 0.15 | |
|  | Medium-Tissue | 4.8 | |
| Maximum cellular uptake rate (mM/S) | Oxygen | 2.07 | |
|  | Glucose | 1.09 | |
| Michaelis-Menten constant (mM) | Oxygen | 4.63x10^-3^ | |
|  | Glucose | 4x10^-2^ | |
| **PCD working conditions** | | | |
| Injection pressure (Pa) | 4 | | |
| Aspiration pressure (Pa) | 4 | | |
| Injection/Aspiration flowrate (nL/s) | 100 | | |

1 Rousset, N., Monet, F. & Gervais, T. Simulation-assisted design of microfluidic sample traps for optimal trapping and culture of non-adherent single cells, tissues, and spheroids. *Scientific reports* **7**, 245 (2017).

2 Place, T. L., Domann, F. E. & Case, A. J. Limitations of oxygen delivery to cells in culture: An underappreciated problem in basic and translational research. *Free Radical Biology and Medicine* **113**, 311-322 (2017).

3 Ariga, O., Kubo, T. & Sano, Y. Effective diffusivity of glucose in PVA hydrogel. *Journal of fermentation and bioengineering* **78**, 200-201 (1994).

4 Figueiredo, L. *et al.* Assessing glucose and oxygen diffusion in hydrogels for the rational design of 3D stem cell scaffolds in regenerative medicine. *Journal of tissue engineering and regenerative medicine* **12**, 1238-1246 (2018).

5 Markov, D. A., Lillie, E. M., Garbett, S. P. & McCawley, L. J. Variation in diffusion of gases through PDMS due to plasma surface treatment and storage conditions. *Biomedical microdevices* **16**, 91-96 (2014).

6 Chowdhury, S., Bhethanabotla, V. R. & Sen, R. Measurement of oxygen diffusivity and permeability in polymers using fluorescence microscopy. *Microscopy and Microanalysis* **16**, 725-734 (2010).

7 Hicks, K. *et al.* An experimental and mathematical model for the extravascular transport of a DNA intercalator in tumours. *British journal of cancer* **76**, 894-903 (1997).

8 Pruijn, F. B., Patel, K., Hay, M. P., Wilson, W. R. & Hicks, K. O. Prediction of tumour tissue diffusion coefficients of hypoxia-activated prodrugs from physicochemical parameters. *Australian journal of Chemistry* **61**, 687-693 (2008).

9 Abaci, H. E., Truitt, R., Tan, S. & Gerecht, S. Unforeseen decreases in dissolved oxygen levels affect tube formation kinetics in collagen gels. *American Journal of Physiology-Cell Physiology* **301**, C431-C440 (2011).
